# High expression of ABCG2 is associated with chemotherapy resistance of osteosarcoma

**DOI:** 10.1186/s13018-021-02204-z

**Published:** 2021-01-28

**Authors:** Hao Shu, Bin Yuan, Yao Huang, Lei Wang, Bing He, Qi Sun, Luning Sun

**Affiliations:** 1 Department of Orthopedics, Sports Medicine Center, Affiliated Hospital of Nanjing University of Chinese Medicine, Hanzhong Road 115, Nanjing, 210029 China; 2 Department of Pathology, The Affiliated Drum Tower Hospital of Nanjing University Medical School, Nanjing, China

**Keywords:** Osteosarcoma, ABCG2, Chemoresistance, Prognosis

## Abstract

**Objectives:**

Previous studies showed overexpression of ABCG2 in a variety of tumor tissues, which could potentially indicate the probability of chemotherapy resistance. This study aimed to reveal the role of ABCG2 in the development of chemotherapy resistance and the prognosis of osteosarcoma (OS).

**Methods:**

Sixty-eight OS patients were included in this study. Tumor tissues were collected for each patient during surgery. DOX-resistant OS cell lines were induced by consecutive exposure of gradually increasing concentration of DOX to the parental cell lines. Lentivirus was used for the knockdown of ABCG2 in OS cells. Cells were treated with the gradient concentration of DOX, and the viability was assessed by CCK8 assay. Total RNA was isolated from the tumor tissues or tumor cells, and the expression of ABCG2 was analyzed by qPCR. The relationship between ABCG2 expression and clinicopathological characteristics of the patients was analyzed using Student’s *t* test or the Chi-square test. The overall survival time was calculated by the Kaplan-Meier method and analyzed by the log-rank test. *p* < 0.05 was considered statistically significant.

**Results:**

DOX-resistant OS cells were successfully established through continuous exposure to DOX. Forty-eight hours after DOX exposure, the IC 50 value of DOX-resistant HOS cells and DOX-resistant U2OS was 3.5 μM and 3.25 μM, respectively. By contrast, those of the untreated HOS and U2OS cells were 1.15 μM and 0.93 μM, respectively (*p* < 0.01). The mRNA expression level of ABCG2 was significantly increased in DOX-resistant cell lines. The CCK-8 assay showed that the DOX-resistant HOS cells and DOX-resistant U2OS cells transfected with ShABCG2 were more sensitive to the DOX treatment than those transfected with ShCtrl. Analysis of gene expression in OS tissues showed remarkably higher expression of ABCG2 as compared with adjacent normal tissues (*p* < 0.01). Patients with high expression level of ABCG2 had obviously decreased overall survival time than the patients with normal expression (*p* < 0.01).

**Conclusions:**

ABCG2 expression level was significantly associated with the resistance to chemotherapy and the overall survival of OS patients. ABCG2 may be a promising therapeutic target for OS patients.

**Supplementary Information:**

The online version contains supplementary material available at 10.1186/s13018-021-02204-z.

## Introduction

Osteosarcoma (OS) is the most common malignant bone tumor characterized by a high incidence of distant metastasis [1, 2]. Before the advent of neo-adjuvant chemotherapy, OS patients undergoing amputation or reconstruction surgery had low 5-year survival rates averaging approximately 15–20% [3, 4]. Benefited from the multi-agent neo-adjuvant chemotherapy, the 5-year survival rate now rises up to 70% for patients with non-metastatic OS [5]. However, it is noteworthy that patients with poor survival rate showed worse responsiveness to chemotherapy. In the past years, different approaches have been applied to investigate the mechanisms involved in chemoresistance of OS [6, 7]. It was speculated that intrinsic gene expression differences may account for the low responsiveness to chemotherapy, while the precise underlying mechanisms remain poorly understood. Herein, identification of genes associated with chemoresistance is of great importance to personalize an effective regimen of chemotherapy.

First cloned from doxorubicin-resistant breast cancer cells, the human ABCG2 gene encodes ATP binding cassette (ABC) transporters that drive the transport of various substrates across cell membranes [8, 9]. Previous studies showed that ABCG2 plays an important role in the proliferation and differentiation of stem cells [10, 11]. Moreover, overexpression of ABCG2 has been observed in a variety of tumor tissues, which could potentially indicate the probability of chemotherapy resistance [12, 13]. Noguchi et al. [13] reported that ABCG2 was related to drug resistance in the breast cancer. Damiani et al. observed that overexpression of ABCG2 could significantly affect the duration of complete remission, which therefore might be regarded as a prognostic factor in patients with acute myeloid leukemia [12].

To our knowledge, studies concerning the role of ABCG2 in OS chemoresistance are still limited. Yet, few studies have investigated the regulatory mechanism underlying ABCG2 expression in OS tissues. In this study, we aimed to investigate the role of ABCG2 in the development of doxorubicin (DOX) resistance and the prognosis of OS. Moreover, we investigated the association of genetic polymorphism with tissue expression of ABCG2 and the chemotherapy resistance of OS patients.

## Methods

### Subjects

Under the approval by the local institutional Ethics Committee, 68 OS patients undergoing treatment in our clinic centers between 2008 March and 2013 January were included in this study. All the patients were histologically diagnosed as OS. Baseline characteristics of the patients were collected from the medical record, including age, gender, tumor size, histologic differentiation, and Enneking stages. Before the surgery, all the patients had received two cycles of neoadjuvant chemotherapy composed of DOX, methotrexate, and cisplatin. Tumor tissues were collected from each patient during surgery. Good response to the chemotherapy was histologically defined as more than 90% necrosis rate in the tumor tissues. All patients signed written informed consent concerning the collection of blood and tissue samples.

### Cell culture and establishment of DOX resistance

The human OS cell lines U2OS and HOS were purchased from the American Type Culture Collection (ATCC, Rockville, MD, USA). All cell lines were cultured in Dulbecco’s modified Eagle’s medium (4.5 g/l glucose)/Ham F12 (1:1) (Invitrogen, Carlsbad, CA, USA) supplemented with 10% fetal calf serum (FCS). All cells were cultured at 37 °C in a humidified atmosphere of 5% CO_2_. DOX was purchased from Sigma (Buchs, Switzerland). DOX-resistant cell lines were induced by consecutive exposure of gradually increasing concentration of DOX to the parental cell lines for 6 months as previously reported. As a starting concentration, 0.015 μM DOX was applied, and the concentration was gradually increased up to 0.12 μM in a period of 3 months. One month after exposure to 0.12 μM doxorubicin, the resistance stability was defined as not significantly altered IC50 of U2OS and HOS cells when incubated in DOX for a week.

### Knockdown of ABCG2 in OS cells

The lentivirus shRNA specifically targeting ABCG2 (shABCG2) and the negative control scramble shRNA (shCtrl) were designed and constructed by Shanghai Genechem Company Ltd., China. Lentivirus particles were generated by co-transfecting recombined and packing vectors into 293T cells via Lipofectamine 2000 (Invitrogen, Carlsbad, CA, USA). U2OS and HOS cells were then cultured in 6-well plates and transfected with shABCG2 or shCtrl. Both cells lines were cultured for 5 days. The knockdown efficiency of the target gene was further evaluated for both cell lines with qPCR analysis and western blot (WB).

### Cell proliferation assay

Cell proliferation was analyzed by the colorimetric water-soluble tetrazolium salt assay using a Cell Counting Kit-8 (CCK8) according to the manufacturer’s instructions (Dojindo, Tokyo, Japan). Cells were treated with the gradient concentration of DOX (0, 0.5, 1.0, 2.0, 4.0, 8.0 μM) for 24 h, and then cultured for another 48 h. CCK-8 reagent (10 μL) was added to each well, followed by incubation for 1 h in a humidified atmosphere (37 °C, 5% CO_2_). The number of viable cells was evaluated by the absorbance at 450 nm using a microplate reader (Tecan Spectra Fluor Plus, Crailsheim, Germany), which was then repeated with 3 replicates.

### qPCR

Total RNA was isolated from the tumor tissues or tumor cells using TRIzol reagent (Invitrogen, Carlsbad, CA, USA) according to the manufacturer’s protocol. Reverse transcription into cDNA was performed with a SuperScript III Reverse Transcriptase kit (Invitrogen, Carlsbad, CA, USA). The quantitative gene expression level was measured using SYBR Master Mixture (TAKARA, Tokyo, Japan) on the LightCycler 480 (Roche Applied Science, Mannheim, Germany). Glyceraldehyde 3-phosphate dehydrogenase (GAPDH) was used as the internal control. The sequences of the primers were as follows: ABCG2, 5′- GCCACAGAGATCATAGAGCCT -3′, 5′- TCACCCCCGGAAAGTTGATG -3′; GAPDH, 5’- GAGTCAACGGATTTGGTCGT -3’, reverse 5’ -TTGATTTTGGAGGGATCTCG- 3’. Quantitative analysis normalized to GAPDH was performed using the 2^−ΔΔCt^ method. All the experiments were performed with 3 replicates.

### Western blot analysis

Cell lysis buffer was used for the preparation of total cell proteins. Cell lysates were separated equally on a 10–12% SDS polyacrylamide gel, which was then electro-transferred to polyvinylidene fluoride membranes (Immobile P; Millipore) and blocked with 5% nonfat dry milk in TBST for 1 h. Membranes were immunoblotted with the primary antibody (Abcam) overnight at 4 °C, and then incubated with second antibody (Cell Signaling Technology) for 2 h at room temperature. The signals were detected by enhanced chemiluminescence (Thermo) with GAPDH used as a loading control.

### Immunohistochemical staining

Paraffin-embedded tissue samples were cut into 4-μm sections and deparaffinized for immunohistochemical staining (IHC). Slides were then incubated with primary anti ABCG2 antibody (1:400, Abcam) overnight at 4 °C, followed by incubation with secondary antibody for 30 min at 20 °C. All slides were stained by diaminobenzidine and then counterstained by hematoxylin. The IHC-stained tissue sections were scored by two pathologists. The signal intensity was scored as follows: score 0, no signal; score 1, weak; score 2, moderate; and score 3, marked. The staining distribution was scored as follows: score 0, no positive staining cells; score 1, 1–30% positive staining cells; score 2, 31–60% positive staining cells; score 3, 61–100% positive staining cells. The final score was calculated by multiplying the score of staining distribution with the signal intensity score. Remarkably higher staining of ABCG2 was defined as the score more than 2.

### Genotyping of functional variant

DNA was extracted using the DNA extraction kit (QIAGEN Inc., Tokyo, Japan) according to the protocol of the manufacturers. SNP rs2231142 (c.421C > A) was genotyped using TaqMan SNP Genotyping Assay. The flanking amplified sequence for the genotyping analysis was as follows: CACCATGGATTAGGAGATCATCAGAGGTC[A/C]CGGACCCCTCTCTGTTACTGAGACATTTG. The genotyping assay was performed with ABI 7900HT Sequence Detection System (Applied Biosystems, Foster City, CA).

### Statistical analysis

The results were presented as mean ± standard deviation and analyzed with the SPSS software (version 19.0; SPSS Inc, Chicago, IL, USA). The data was shown as means ± SD for continuous variables. The relationship between ABCG2 expression and clinicopathological characteristics of the patients was analyzed using Student’s *t* test or the Chi-square test. The gene expression and the overall survival were compared among different genotypes of rs2231142 with one-way ANOVA test. The overall survival time was calculated by the Kaplan-Meier method and analyzed by the log-rank test. *P* < 0.05 was considered statistically significant.

## Results

### Demographic data

The demographic and clinical characteristics of the study subjects are shown in Table 1. The mean age of patients with OS was 35.2 ± 16.1 years. Thirty patients were male, and 38 patients were female. Fifty-seven (83.8%) patients were at tumor stages I–II. Fifty-four (78.4%) patients received limb salvage treatment. After surgery, 27 (39.7%) patients were found to have good response to the chemotherapy. After a mean period of 39.5 months (range, 8–60 months) follow-up, 32 (47.1%) patients had died from all causes.

**Table 1:** Baseline characteristics of the patients

Features	Patients (*n* = 68)
Gender	
Male	30
Female	38
Age (years)	
> 20	34
≤ 20	34
Enneking stages	
I	13
IIA	17
IIB	27
III	11
Histologic type	
Osteoblastic	35
Chondroblastic	17
Fibroblastic	2
Mixed	14
Tumor size (cm)	
> 5	41
≤ 5	27

### ABCG2 was overexpressed in DOX-resistant OS cells

**Fig. 1:**
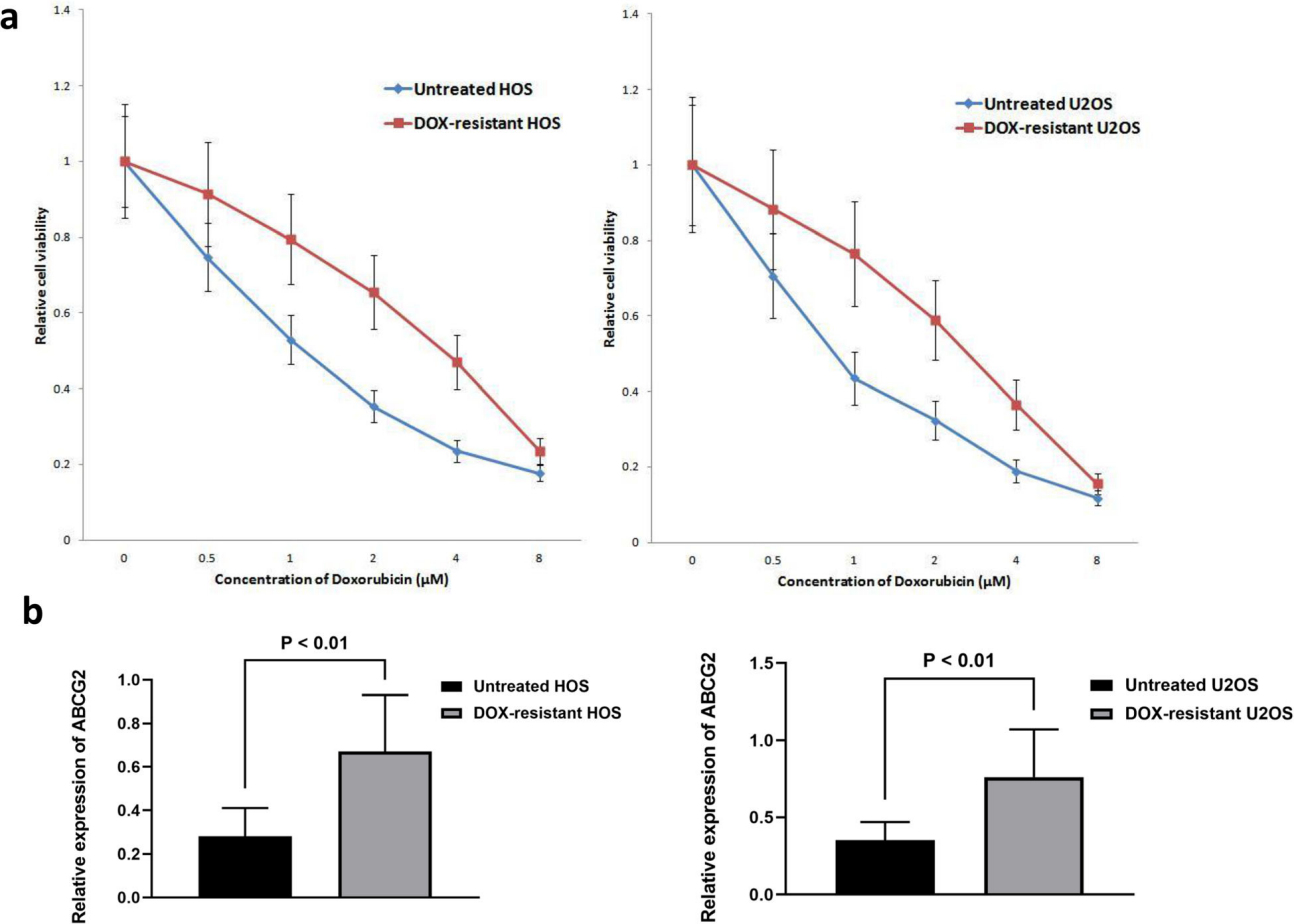
Upregulated expression of ABCG2 in DOX-resistant OS cells. **a** Successful establishment of DOX-resistant OS cell lines was verified by CCK8 assay. Cell viability of DOX-treated HOS cells and U2OS cells was remarkably higher than those not treated by DOX. **b** Through qPCR, we confirmed that mRNA expression level of ABCG2 was significantly increased in DOX-resistant cell lines as compared with normal cell lines

Figure 1 showed that DOX-resistant OS cells were successfully established through continuous exposure to DOX. Forty-eight hours after DOX exposure, the IC 50 value of DOX-resistant HOS cells and DOX-resistant U2OS were 3.5 μM and 3.25 μM, respectively. By contrast, those of the untreated HOS and U2OS cells were 1.15 μM and 0.93 μM, respectively (*p* < 0.01). The results of qPCR analysis showed that mRNA expression level of ABCG2 was significantly increased in DOX-resistant cell lines (Fig. 1).

### Knockdown of ABCG2 enhanced response to DOX in OS cells

**Fig. 2:**
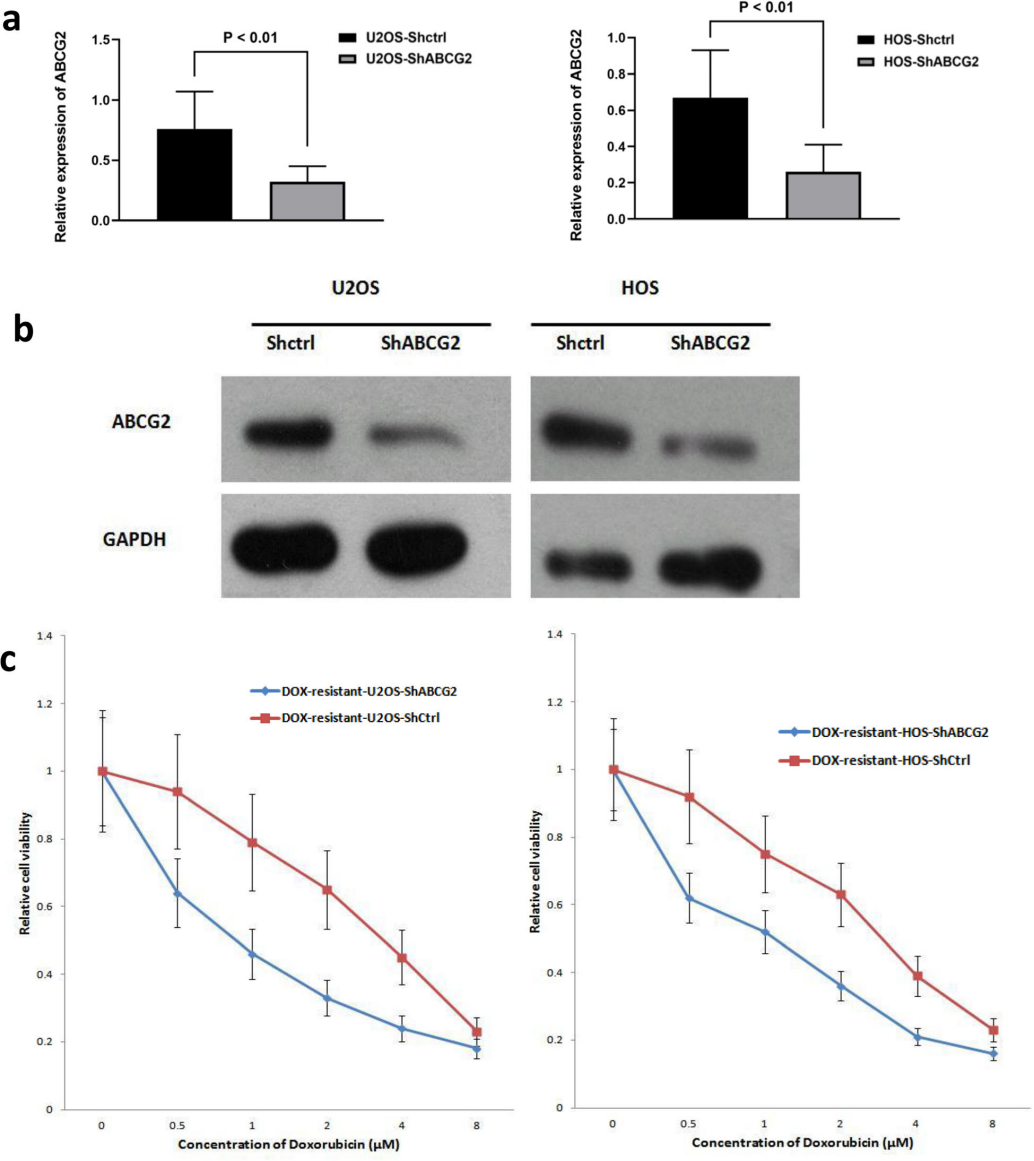
Effect of knockdown of ABCG2 on the sensitivity of OS cell lines to DOX. **a**, **b** ABCG2 mRNA expression in OS cell lines was effectively knocked down by lentivirus as evidenced by qPCR and WB analysis. **c** Knockdown of ABCG2 could reverse the resistance of OS cells to DOX. The CCK-8 assay showed that the DOX-resistant HOS cells and DOX-resistant U2OS cells transfected with ShABCG2 were more sensitive to the DOX treatment than those transfected with ShCtrl

As shown in Fig. 2, the mRNA and protein expression level of ABCG2 were remarkably decreased in both ABCG2-inhibited HOS and U2OS cells as compared with the control cells. After being incubated with DOX for 48 h, the viability of cells transfected with shABCG2 was remarkably lower than that of cells transfected with shCtrl. The IC 50 value of ShABCG2-transfected HOS cells and ShABCG2-transfected U2OS was 3.25 μM and 3.05 μM, respectively. By contrast, the IC 50 value of HOS and U2OS cells in the control group was 1.05 μM and 0.97 μM, respectively (*p* < 0.01).

### High expression level of ABCG2 was associated with a poor prognosis of OS

**Fig. 3:**
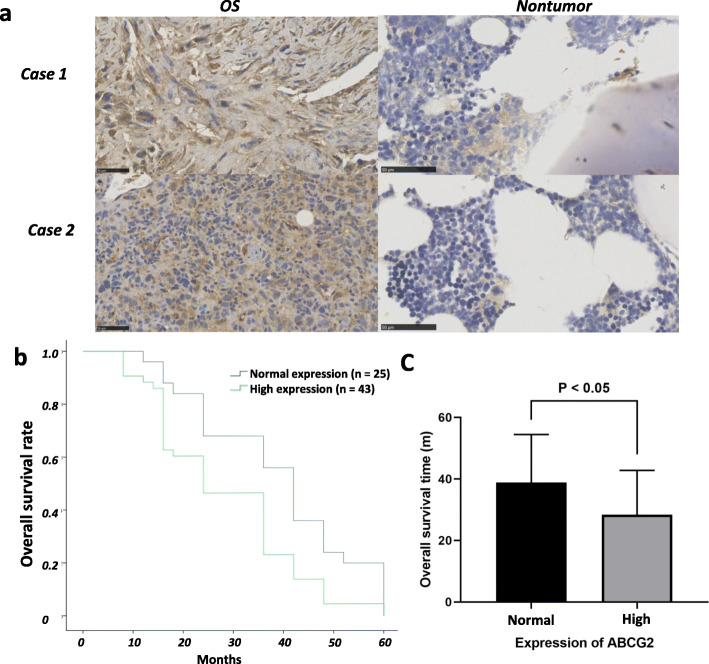
Relationship between ABCG2 expression and the prognosis of OS patients. **a**. Analysis of gene expression in OS tissues showed remarkably higher expression of ABCG2 as compared with adjacent normal tissues (*p* < 0.01). **b**, **c** Patients with high expression level of ABCG2 had obviously decreased overall survival rate and lower survival time than the patients with normal expression of ABCG2 (*p* < 0.05)

The ABCG2 expression was analyzed in the tumor tissues and adjacent normal tissues of 68 patients. As shown in Fig. 3, ABCG2 was obviously highly expressed in the tumor tissues than in the normal tissues (*p* < 0.001). According to the results of IHC, 43 patients were included in the high expression group and the other 25 patients were included in the normal expression group (Supplementary Figure 1). As shown in Table 2, we found that ABCG2 expression was significantly associated with tumor size (6.1 cm ± 2.7 cm vs. 3.9 cm ± 1.5 cm, *p* = 0.01) and the response to chemotherapy (62.4% ± 25.1% vs. 75.3% ± 18.7%, *p* = 0.02). As shown in Fig. 3, the overall survival time of patients with normal ABCG2 expression was significantly longer than those of the high ABCG2 expression (38.9 ± 15.6 months vs. 28.4 ± 14.4 months, *p* < 0.01). No significant association was found between ABCG2 expression and other clinical features (Table 2).

**Table 2:** Relation between ABCG2 expression and clinical features of the patients

	ABCG2 expression		*p*
	Normal expression (*n* = 25)	High expression (*n* = 43)	
Age (years)			
> 20	13	21	0.65
≤ 20	12	22	
Mean ± S.D.	34.5 ± 15.7	35.6 ± 14.9	0.77
Gender			
Male	11	19	0.98
Female	14	24	
Enneking stages			
I	4	9	0.35
IIA	7	10	
IIB	12	15	
III	2	9	
Tumor size (cm)	3.9 ± 1.5	6.1 ± 2.7	< 0.01
Overall survival (month)	38.9 ± 15.6	28.4 ± 14.4	< 0.01

### SNP rs2231142 might play a functional role in the ABCG2-asociated prognosis of OS

**Fig. 4:**
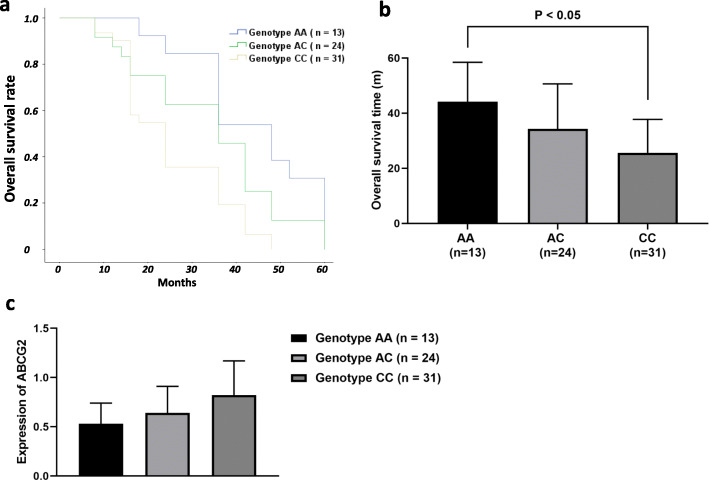
Role of rs2231142 in the ABCG2-associated prognosis of OS. **a**, **b** Patients carrying the genotype AA were observed to have significantly increased overall survival rate and longer overall survival time than those carrying genotype CC (*p* < 0.05) **c** Genotype AA of rs2231142 was indicative of remarkably lower expression of ABCG2 than genotype CC (*p* < 0.01)

Genotyping of rs2231142 was successfully completed for all the patients. Of the 68 patients, there were 31 patients with genotype CC, 24 with genotype CA, and 13 with genotype AA. The mRNA expression of ABCG2 in tumor tissues was analyzed for each patient. As shown in Fig. 4, patients carrying genotype AA had remarkably lower expression of ABCG2 than those with genotype CC (0.53 ± 0.21 vs. 0.82 ± 0.35, *p* < 0.01). Moreover, patients carrying the genotype AA were associated with longer overall survival time as compared with those with genotype CC (44.1 ± 14.3 months vs. 25.6 ± 12.1 months, *p* < 0.01) (Fig. 4).

## Discussion

Chemotherapy regimen composed of DOX, methotrexate, and cisplatin is most commonly applied for patients with OS [5, 14]. However, resistance to chemotherapeutic drugs remains the primary obstacle to achieve favorable outcome of human cancer. Various factors leading to chemoresistance have been reported in previous studies, such as alterations in the drug target, abnormal induction of cell death pathways, and overexpression of ABC membrane transporter family members [15–17]. Nevertheless, there is still a lack of methods to predict chemoresistance in OS effectively. Obviously, identifying new contributing factors of chemoresistance in OS is of great importance to improve the therapeutic efficacy and predict the drug response.

High ABCG2 expression has been identified in a variety of solid tumors and has been correlated with poorer clinical outcomes [8–10]. In a recent study, Tsai et al. [18] reported that ABCG2 overexpression could decrease the therapeutic effect of DOX in OS cells. For the first time, we confirmed the role of ABCG2 in the development of chemoresistance using the established DOX-resistant OS cell lines. Comparably, through Genechip analysis, Walters et al. [19] identified increased expression of ABCG2 in the OS cell lines treated by doxorubicin. Compared with their parental DOX-sensitive cells, DOX-resistant cells were observed to have remarkably elevated expression level of ABCG2. Furthermore, knockdown of ABCG2 level in DOX-resistant cell lines by lentivirus could effectively recover the therapeutic efficacy of DOX treatment. Of note, we found that ABCG2 expression level was significantly increased in patients with decreased overall survival and poor response to chemotherapy. It was in accordance with previous study reporting that elevated levels of ABCG2 have a vital role in OS prognosis [20]. On the basis of these data, we proposed that ABCG2 may become a promising therapeutic target for OS patients. These characteristics of ABCG2 enable it a promising molecular target to guide the chemotherapy treatment of OS.

The genetic variants of ABCG2 were reported to be involved in cancer risk and chemotherapeutic response [21, 22]. To illustrate the mechanism underlying the regulation of ABCG2 expression in OS tissues, a functional polymorphism rs2231142 of ABCG2 was genotyped in our cohort of patients. We confirmed that genotype CC of rs2231142 was indicative of remarkably higher expression of ABCG2. For OS patients receiving a therapeutic program that includes DOX, those with genotype CC could have poor outcome, which was in line with our finding that high expression of ABCG2 was associated with lower survival rate. Comparably, Morisaki et al. [23] found that cells transfected with the allele C of rs2231142 showed remarkably higher resistance to chemotherapy drugs than those transfected with wild-type, suggesting that rs2231142 may affect drug transport. Additionally, the rs2231142 has also been reported to be associated with increased adverse effects in response to chemotherapy treatment. Mizuarai et al. [24] found that rs2231142 mutant may decrease ATPase activity by 1.3-fold as compared with the wild-type ABCG2. Taken together, these results suggested that rs2231142 may result in altered transport functions of ABCG2 transporter, which has important implications for the pharmacokinetics and drug-resistance profiles of chemotherapeutics. To conclude, we have provided the first data concerning the impact of ABCG2 polymorphism in the prognosis of patients with OS. Allele C could be the clinical relevant allele which was indicative of decreased response to chemotherapy and poor prognosis. Early detection of this variant could be helpful in driving the choice of other alternative drugs to be used in chemotherapy.

Several limitations of this study should be addressed here. Due to the inherent drawback of retrospective study, the sample size of the OS patients was relatively small. In the future study, more OS patients need to be recruited for a stronger statistical power. Second, the mechanism underlying the influence of rs2231142 on expression level of ABCG2 remains undetermined, which is worthy of further investigation in the future study. Third, more in vitro experiments are warranted to determine the role of methotrexate and cisplatin in ABCG2-associated chemotherapy resistance.

## Conclusions

ABCG2 expression level was significantly associated with the response to chemotherapy and the overall survival of OS patients. Allele C of rs2231142 was indicative of higher ABCG2 expression and associated with poor prognosis of OS. ABCG2 may become a promising therapeutic target for OS patients. Presence of allele C of rs2231142 could suggest the choice of other alternative drugs to be used in chemotherapy.

## Supplementary Information

**Additional file 1: Supplementary Figure 1.** Representative cases with high tissue expression of ABCG2. 4 representative cases were selected to present the IHC staining of ABCG2 in OS tissues. Case # 08 and case # 11 had strong signal intensity and positive staining cells, who were assigned to high expression group. By contrast, case # 06 and case # 09 had weak signal intensity and less positive staining cells, who were then assigned to normal expression group.

## Data Availability

All data used in this study are available at the request of editors, reviewers, and the research community.

## Abbreviations

OS: Osteosarcoma
ABC: ATP binding cassette
DOX: Doxorubicin
CCK8: Cell Counting Kit-8
