# Supplementary figures and images for: High expression of ABCG2 is associated with chemotherapy resistance of osteosarcoma
Source: J Orthop Surg Res. 2021 Jan 28;16:85. doi: 10.1186/s13018-021-02204-z (PMC7842061; doi:10.1186/s13018-021-02204-z)

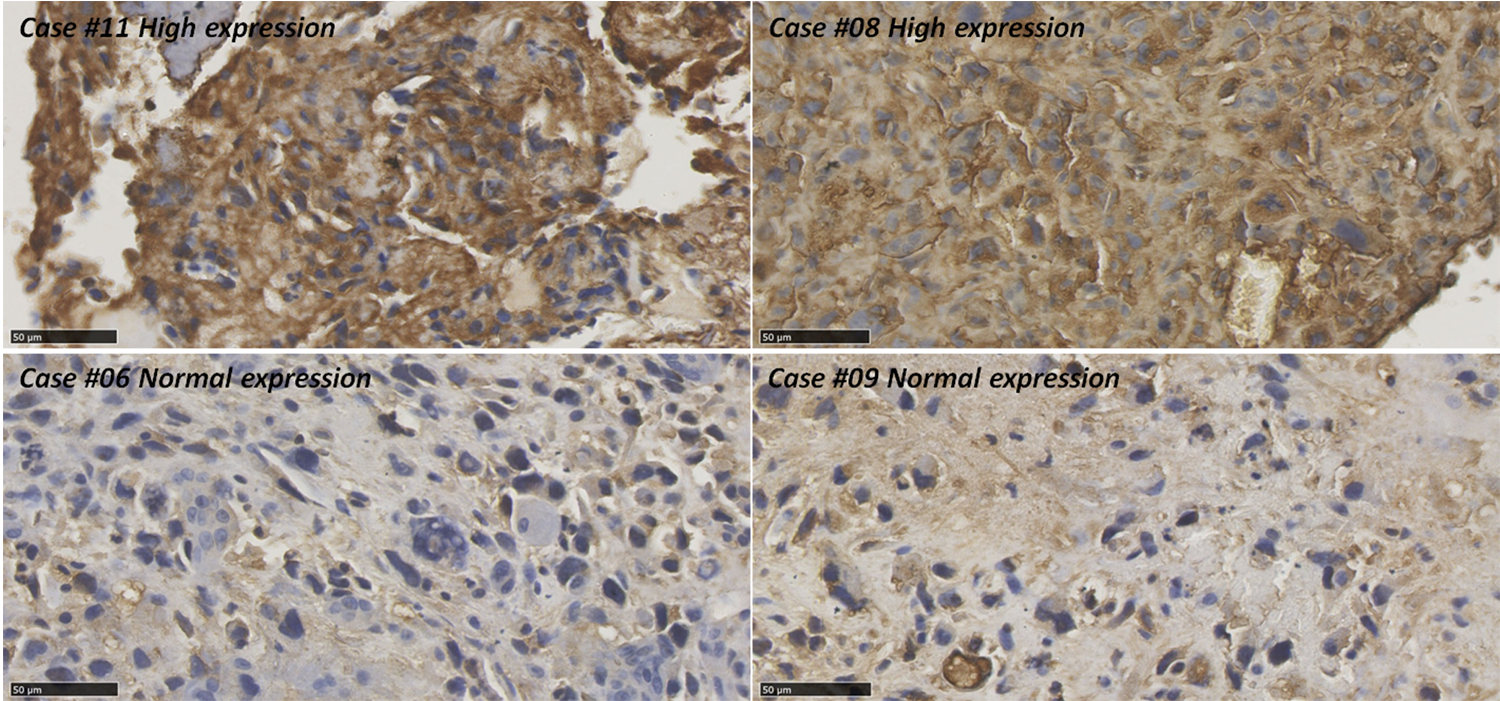

Supplement: Supplementary file 1 — Additional file 1: Supplementary Figure 1. Representative cases with high tissue expression of ABCG2. 4 representative cases were selected to present the IHC staining of ABCG2 in OS tissues. Case # 08 and case # 11 had strong signal intensity and positive staining cells, who were assigned to high expression group. By contrast, case # 06 and case # 09 had weak signal intensity and less positive staining cells, who were then assigned to normal expression group. [file 13018_2021_2204_MOESM1_ESM.tif]
